# Current Strategies for Perinatal Mental Health Care in Japan

**DOI:** 10.31662/jmaj.2023-0093

**Published:** 2024-01-15

**Authors:** Shunji Suzuki

**Affiliations:** 1 Department of Obstetrics and Gynecology, Japanese Red Cross Katsushika Maternity Hospital, Tokyo, Japan

**Keywords:** perinatal mental health care, multidisciplinary collaboration, Japan, preconception care, obstetric institute

## Abstract

Perinatal mental health care is required to maintain the emotional well-being of pregnant women, as well as their children, partners, and families. The mental and physical support for the pregnant and/or postpartum women with serious mental health problems should be provided with multidisciplinary collaboration in the perinatal area. The adverse outcomes related to impaired perinatal mental health are suicide and child abuse, which are the top reasons why mental health care in pregnant and/or postpartum women is important. Mental health care during the perinatal period should be provided proactively with interventions from medical practitioners. In addition, to promote the provision of information on health management for future pregnancies, “preconception care” through consultation, supported with medical examinations, is also important.

## Introduction

Perinatal mental health problems can functionally impair pregnant and/or postpartum women and are associated with the suboptimal development of their children ^(1), (2), (3), (4)^. For example, previous animal investigations have linked antenatal stress with a range of persistent behavioral abnormalities in the offspring ^(2)^. In addition, antenatal stress and anxiety and mood disorders have been observed to have a programming effect on the fetuses and their brain development at least until middle childhood ^(2), (3), (4)^. The adverse outcomes related to impaired perinatal mental health are suicide and child abuse, which are also one of the outcomes of untreated maternal mental health disorders, in addition to serious social and physical problems that include murder-suicide or child neglect by mothers ^(5), (6), (7)^. Furthermore, early severe stress and maltreatment have been reported to produce a cascade of neurobiological events that have the potential to cause enduring changes in child brain development ^(8), (9)^. These serious perinatal outcomes associated with mental health problems may be rare; however, a 2006 multicenter study on the epidemiology of perinatal depression in Japan reported that the incidence rate of the onset of depression during pregnancy and within 3 months after delivery is 5.6% and 5.0%, respectively ^(10)^. In addition, antenatal depression seemed to be a major risk factor for postnatal depression ^(10)^. Therefore, perinatal mental health care is required to maintain the emotional well-being of pregnant women, as well as their children, partners, and families.

In Japan, the Basic Law for Child and Maternal Health and Child Development, which a philosophical law enacted in 2018 ^(11)^, has aimed to comprehensively promote measures to provide necessary medical care for growing children and their guardians as well as pregnant and/or postpartum women in a seamless manner. The care for the “Child and Maternal Health and Child Development” requires services associated with medical care, health, education, and welfare to comprehensively and appropriately address the problems related to pregnancy, childbirth, and child-rearing. In the law, to promote the provision of information on health management for future pregnancies, “preconception care” through consultation, supported with medical examinations, is also important ^(11), (12)^. Preconception care will also promote a healthy development process for mothers and children, emphasizing their relationship during the period from pregnancy to postpartum childcare.

In 2010, due to concerns about inappropriate parenting attitudes and behaviors, the Japanese Ministry of Health, Labour and Welfare defined “specified expectant mothers” (SEMs) as pregnant women at high risk of abuse and in need of extra support after birth because of some medical and/or social problems such as having an unstable income and mental health disorders, to name a few ^(13)^. In many institutes in Japan, during pregnancy, midwives or obstetricians usually conduct health consultations of pregnant women and obtain information on their mental health status using a simple questionnaire ^(14), (15)^. However, in the event that midwives, obstetricians, or clinical psychologists are unable to resolve their mental health problems, a psychiatrist is consulted for diagnosis and therapy.

Based on these backgrounds and the viewpoints of the law ^(11)^, this study reviews the current outlines of the physical and mental health of mothers and children.

## Mental and Physical Care from Birth (Preconception) to Adulthood

The “development process” will mean the series of mental and physical growth processes from birth to adulthood ^(11)^. This is one of the preambles of the Basic Law for Child and Maternal Health and Child Development. In this law, the healthy mental and physical development of children in the next generation from preconception to adulthood is marked as the responsibility of the entire nation of Japan. Because a positive association has been observed between maternal neurological or psychiatric disorders during pregnancy and adverse perinatal outcomes ^(16)^. Untreated maternal mental health disorders are identified as a risk factor itself. Depressive symptoms during pregnancy have also been observed to be associated with early life stress with major changes ^(17)^. In addition, multidisciplinary efforts must be made to provide perinatal mental and physical support for pregnant and/or postpartum women ^(18)^. In many institutes in Japan, multidisciplinary collaboration is composed of midwives, clinical psychologists, medical social workers, medical treasurers, and regional administrative staff ^(14), (19)^. Clinical psychologists evaluate the mental health status of patients and refer them to psychiatrists and/or regional staff when necessary. The medical social workers provide support and information on the available social resources and serve as contact points for regional organizations. The information as regards this group of pregnant women is then shared, wherein opinions from each specialized area are exchanged and support methods are decided. Specifically, a clinical system or community medical cooperation system must be established, including enhancement of the guidelines and/or manuals related to perinatal basic and basic and emergent collaboration in health care.

In the perinatal area, it is important to properly evaluate the mental health of pregnant women, prevent perinatal depression and anxiety and child abuse, and establish a healthy mother-child relationship ^(12)^. Furthermore, considering mothers’ anxieties associated with child-rearing, it is necessary to examine a continuous prenatal and postnatal mental health care system for these mothers to experience the enjoyment and fulfillment of child-rearing while providing social resources (or the information of social resources). In addition, educational opportunities should be provided through prenatal classes at perinatal facilities to promote partners’ participation in childcare ^(12)^. At these venues, all pregnant women and their partners are made aware regarding the possible occurrence of perinatal mental health disorders and that early diagnosis and treatment are necessary and effective ^(18)^. In order to promote parent-child relationships, a support center such as the “Comprehensive Support Center for Child-rearing Generations” for pregnant and/or postpartum women that can organically integrate existing support systems should be established ^(18)^. In addition, obstetricians, pediatricians, psychiatrists, and related collaborating staff, including local government officials, should be present to provide comprehensive support covering the prenatal, postnatal, and childcare periods ^(18)^.

## Preconception Care and Shared Decision-making (SDM)

Stabilization of the mental state during prepregnancy has been reported to prevent the onset and deterioration of postpartum mental health disorders. Therefore, preconception care has been recommended to adjust women’s mental condition before pregnancy ^(12), (20)^. Preconception care is provided when a patient and her partner or family with a mental health disorder or a history of a mental health disorder consult about future pregnancies. For example, the dosage of psychiatric medicines should be adjusted for pregnancy ^(21)^. In addition, the pregnant woman should be provided with assistance in building a good relationship with her family and those around her ^(22), (23)^. If the interventions cannot be provided prior to pregnancy or if the consultations are made after pregnancy, the interventions should be started as soon as possible. If the facility finds it difficult to provide sufficient support to the patient, cooperation with other facilities that provide the relevant care is desirable ^(12), (20)^. From the perspective of perinatal mental health, it is ideal to aim for pregnancy after confirming that the mental state of the woman is stable for about 3-6 months; however, we should note the possible situation that women with mental health problems sometimes cannot tell their desire to conceive ^(18)^. In addition, when pregnancy does occur, there is not guarantee that their obstetricians are made aware of their mental health conditions. Therefore, there are some cases of pregnancy without sufficient information, voluntary discontinuation of medications due to the fear of its effects on the fetus, and/or preventing psychiatric hospital visits leading to the worsening of prenatal and postnatal mental health disorders, leading to undesirable results. To prevent the risk of mental health disorders in pregnant women, it is important to support their mental health by telling them that the use of medications prescribed as necessary actually promotes the growth and development of the fetus.

Ultimately, SDM will be very important after fully sharing and considering the risks and benefits of medication from an early stage of prepregnancy that fully considers the individuality of each woman and family ^(12), (20)^. While it is necessary to share and consider options for contraception until the stabilization of mental health disorders to avoid unexpected pregnancies, the risk of perinatal complications increases as maternal age increases ^(24), (25)^. Therefore, appropriate SDM is important in considering the balance of the lifelong physical and mental health of women and children. In addition, we should also remember to prepare for “interconception care” in cases of unexpected pregnancies.

## Necessity of a Multidisciplinary Collaboration

Pregnant women requiring mental health support at perinatal medical institutions are those who are assumed to have a poor childcare environment before birth, which includes (1) those with mental health disorders such as depression and (2) those with negative feelings toward fetuses and newborns. However, since there are many problems that cannot be covered by medical professionals alone, multidisciplinary collaboration with the staff of the local government agencies is necessary ^(18)^. Local government agencies have a wide range of roles, including environmental conservation, education, welfare, and public health. These include supporting improved living conditions that can affect the mental health and feelings of loneliness and isolation in postpartum families.

## Multidisciplinary Collaboration in Obstetric Institutes

Mental health problems in the perinatal period can occur in anyone; however, some characteristics of the period (early pregnancy and 3-4 months after childbirth) and the risk factors have been observed to be clear to some extent such as unexpected pregnancy, strong anxiety against pregnancy, history of mental disorders, lack of support, and unstable family situation ^(18)^. Furthermore, as mentioned above, we have to keep in mind that women with mental health problems tend to not seek support themselves ^(18)^. Especially those who do not have a history of mental health disorders, they may not be aware of their depressive status. Notably, in Japan, pregnant women without any complaints visit medical institutions for prenatal visits every 2-4 weeks; therefore, medical institutions should have a proactive approach by using these visits to ask pregnant women about possible mental health problems.

In Japan, more than half of all deliveries have taken place in private clinics or small hospitals without the presence of pediatricians or psychiatrists. In the obstetrics institutes of Japan, nurses play the most important role in antenatal mental health care while midwives in particular have a closer relationship with pregnant women and their families. Midwives are in a position where it is easy to first grasp the changes in the mental status of pregnant women and/or their family relationships during prenatal visits, and they can provide the continuous and preventive support through communication skills, which involves listening and accepting the feelings of pregnant women and showing sympathy for them and their feelings. If midwives cannot resolve the problems associated with perinatal mental health disorders, psychiatrists should be consulted, with the midwife still rendering continuous support to prevent feelings of abandonment in pregnant women.

Aside from the multidisciplinary collaboration of healthcare workers in obstetric institutions, from the perspective of medical insurance, it is also necessary to provide opportunities for consultation with psychiatrists, pediatricians, and local administrative staff, as necessary. In addition, psychiatric nurses certified by the Japanese Nursing Association should provide preventive and early intervention for mental health disorders, monitor mental health status, provide psychological interventions such as psychoeducation and cognitive behavioral therapy, and support self-determination about receiving psychotropic medications.

## Guidelines and Workshops for Perinatal Mental Health

A variety of manuals and guidelines have been published in Japan with the aim of promoting mother-infant bonding and attachment formation, and seamlessly supporting healthy parent-child relationships for all pregnant and/or postpartum women ^(12), (18)^. Various guidelines have been published for various occupations according to the level of perinatal mental problems that need to be addressed. The “Clinical Guide for Women with Mental Health Problems during the Perinatal Period” developed in collaboration with psychiatrists and obstetricians is a guideline for a high-risk approach for affected women with mental health problems ^(12)^, while “Perinatal Mental Health Care Manual by the Japan Association of Obstetricians and Gynecologists” is a guideline on how midwives should conduct a population approach under the guidance of a psychiatrist for women in general who do not have mental health problems ^(18)^.

As previously stated, mental health support for pregnant women will require the involvement of multidisciplinary collaboration in the fields of medicine, health, and welfare ^(14), (19)^. Psychosocial interventions have been observed to be effective in preventing common perinatal mental health disorders and fostering positive mental health ^(26), (27)^. In recent years, in order to understand what kind of evaluation should be done and what kind of organization should be cooperated with at what timing to solve the problems that have occurred in the SEM, it has become possible to simulate the step-by-step examination of the points of care by participating in some workshops that are frequently held in Japan in which the impact of perinatal mental health disorders on mothers and their children, the importance of proactive interventions for women with perinatal mental health problems and how to communicate with them, and how to approach the support needed for them are discussed ^(1)^. Because each staff for mental health care needs to have the skills to strive to understand the characters of SEM, it is possible to honing these skills through these workshops ^(1)^.

With recognizing that perinatal mental health problems can develop in anyone, health consultations should be performed to focus on the tone of voice and manner of speaking, how they perceive and understand pregnancy and childcare, and their relationship with their families.

## Social Resources and Community Support for Perinatal Mental Health Care

In addition to medical care, it is important to provide social resources and community support for the changes in women’s mental health during pregnancy, childbirth, and child-rearing those are not seen at other life stages.

The 2017 revision of the Japanese Maternal and Child Health Act have mandated the establishment of the Comprehensive Support Center for Child-rearing Generations in each municipality of Japan ^(18)^. Due to the difficulty of sufficient information sharing and cooperation between medical institutes and local administrative agencies, mental health support has been fragmented by local systems and institutions. The essential duties of the support center are as follows ^(18)^: (1) grasp the actual situation of SEMs and infants, (2) provide consultation on pregnancy, childbirth, and childcare, (3) provide necessary information and health guidance and formulate support plans, and (4) coordinate collaboration with welfare institutions.

In addition, a postpartum care project will be rolled out nationwide by the end of fiscal 2024 to support the physical recovery and psychological stability of mothers so that mothers, children, and their families can raise children in a healthy manner ^(28)^. In the project, nurses and midwives play a central role in promoting the physical recovery and psychological stability of mothers, nurturing mothers’ self-care abilities and encouraging the formation of attachment between mothers and their children. To ensure that mothers with mental health problems can receive high-quality postpartum care anywhere in the country, it is necessary to eliminate regional disparities in subsidies ^(18), (28)^.

In the future, it is hoped that postpartum support will be expanded through the multidisciplinary collaboration led by the Comprehensive Support Center for Child-rearing Generations, which will lead to the prevention of postpartum depression and support raising healthy children.

## Conclusions

In the current opinion, in Japan, mental health care for pregnant women has been outlined from the viewpoint of the Basic Law for Child and Maternal Health and Child Development. Important issues to respect the individual dignity of children who will be responsible for the society of the next generation and to ensure their healthy mental and physical growth need to be addressed. It is also important that all families, not just mothers, receive continuous and comprehensive mental and physical support.

A proactive approach in addressing mental health care during the perinatal period and interventions from medical practitioners are vital to prevent the adverse outcomes related to impaired perinatal mental health such as suicide and/or child abuse.

## Article Information

### Conflicts of Interest

None

### Author Contributions

Shunji Suzuki designed the study and collected the data. In addition, he accepts the responsibility for the entire content of this manuscript and approves of its submission.

### Approval by Institutional Review Board (IRB)

The study protocol was approved by the Ethics Committee of the Japanese Red Cross Katsushika Maternity Hospital (2022-21). The data supporting the results of the current study are available in the article.
